# Tubercular retinal vasculitis mimicking frosted branch angiitis: a case report

**DOI:** 10.1186/s12348-018-0145-8

**Published:** 2018-01-22

**Authors:** Manisha Agarwal, Ankita Shrivastav, Abdul Waris

**Affiliations:** 1 Dr. Shroff’s Charity Eye Hospital, 5027, Kedarnath Road, Daryaganj, New Delhi, 110002 India; 2 Institute of Ophthalmology, Aligarh Muslim University, Aligarh, India

**Keywords:** Tuberculosis, Vasculitis, Vein occlusion, EUS-FNAC

## Abstract

**Background:**

Tubercular vasculitis is an important manifestation of ocular tuberculosis and this report highlights the mimicking nature of the disease with frosted branch angiitis.

**Results:**

A patient presented with a severe form of retinal vasculitis in both eyes and a branch retinal vein occlusion in the left eye. He had a positive tuberculin skin test (TST) and a raised erythrocyte sedimentation rate (ESR) and serum angiotensin-converting enzyme (ACE) levels. Radiological investigations revealed a sub-pleural nodule and mediastinal lymph nodes, which on histopathological evaluation confirmed a granulomatous etiology.

**Conclusion:**

Retinal vasculitis secondary to tubercular etiology may mimic a viral vasculitis; however, a clinical suspicion with a timely diagnosis and management helps in preventing loss of vision and the eye.

## Introduction

Tuberculosis is endemic in Asian countries and often has varied clinical manifestations [1, 2]. It is important to be aware of the various clinical signs and symptoms along with a high index of suspicion for ocular tuberculosis. The confirmation of the diagnosis by a single laboratory test is not available especially in ocular tuberculosis as attaining a sample for laboratory testing is often difficult [3].

We report this case which presented as severe retinal vasculitis due to tubercular etiology resembling frosted branch angiitis secondary to viral etiology. However, a high level of suspicion made us investigate the patient thoroughly including histopathological examination of the sample got by endoscopic ultrasound guided fine needle aspiration cytology (EUS-FNAC), which confirmed the diagnosis and helped in a timely management.

## Case report

A 45-year-old male patient presented with the complaints of gradual painless diminution of vision in the right eye for the last 15 days. There was no history of fever, backache, weight loss, contact, or any other associated systemic illness. The best corrected visual acuity was 6/60, N36 (Snellen’s chart) in the right eye and 6/6, N6 in the left eye.

**Fig. 1:**
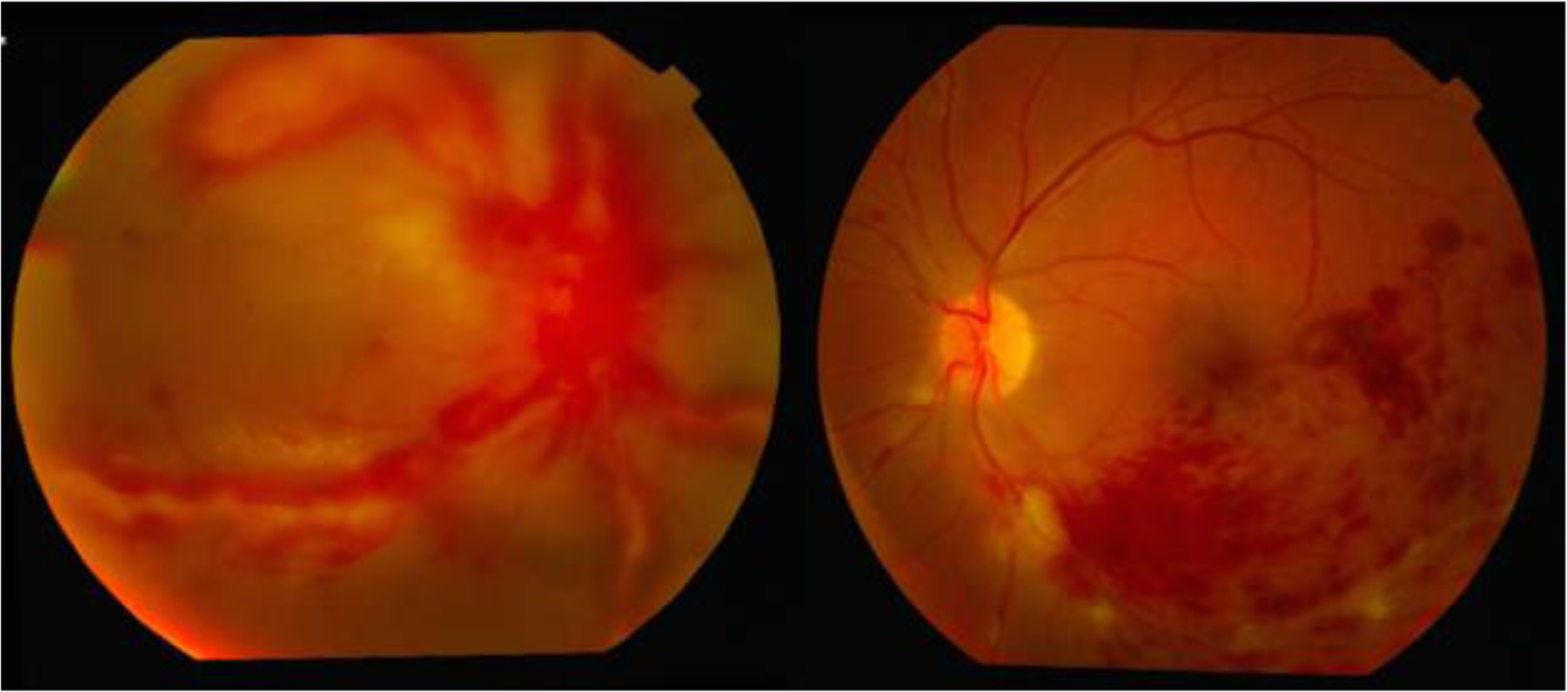
Severe vasculitis involving major vessel arcades in the right eye (left); inferotemporal vein occlusion with hemorrhages in the left eye (right)

Anterior segment examination was within normal limits in both the eyes. Fundus examination of the right eye showed a hyperemic optic disc with severe perivasculitis involving the major vessel arcades with macular edema along with exudation at the posterior pole. The left eye showed an infero-temporal branch retinal vein occlusion with retinal hemorrhages (Fig. 1). Applanation tonometry recorded an intraocular pressure (IOP) of 10 mm Hg (mmHg) in both the eyes.

The patient was investigated including a complete hemogram, which was within normal limits except a raised ESR of 24 mm (mm) in the first hour. An induration of 25 × 25 mm was seen on TST with five purified protein derivative (PPD) units. Immunoglobulin G (IgG) titres for cytomegalovirus (CMV) and herpes simplex virus (HSV) were positive. Immunoglobulin M (IgM) titres for both CMV and HSV were within normal. Serum angiotensin converting enzyme (ACE) levels were raised to 82 μl (μL). On evaluation the patient was not immunocompromised.

Contrast-enhanced computed tomography (CECT) of the thorax showed multiple non-necrotic, non-calcified mediastinal lymph nodes, fibrosis in the right middle lobe and a small sub-pleural nodule in the posterior segment of the right upper lobe.

Transabdominal endoscopic ultrasound (EUS) guided biopsy from the sub-carinal lymph nodes was done and sent for culture and histopathology which showed epithelioid cell granulomas and lymphoid cells with necrosis. Ziehl-Neilsen staining was negative for acid-fast bacilli, and there was no evidence of atypical cells.

Systemic anti-tubercular treatment was initiated with 600 mg of isoniazid, 450 mg of rifampicin, 1200 mg of pyrazinamide, and 750 mg of ethambutol along with oral prednisolone at 1 mg/kg body weight and topical prednisolone acetate (1%) eye drops four times a day.

At 1-month follow up the BCVA in the right eye was 6/12,N8 in the right eye and 6/6,N6 in the left eye. IOP was 16 mm of Hg in both the eyes. Fundus examination of both eyes showed hyperemic optic disc with persistent vasculitis. Fundus fluorescein angiography (FFA) was done which showed leakage from the vessels and late leakage of the disc in both the eyes. The patient was asked to continue the same treatment and follow up monthly.

**Fig. 2:**
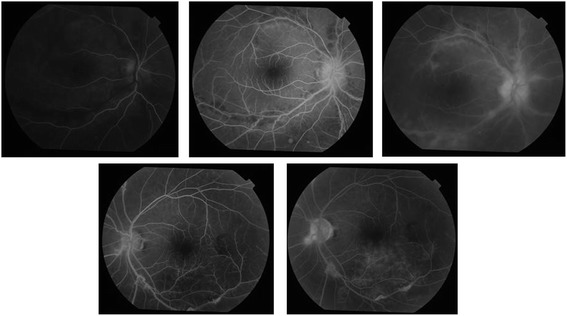
FFA of the right eye (top) shows minimal leakage at the disc with very mild hyperfluorescence along the vessels in late. The left eye (bottom) shows minimal disc leak with resolving vein occlusion

**Fig. 3:**
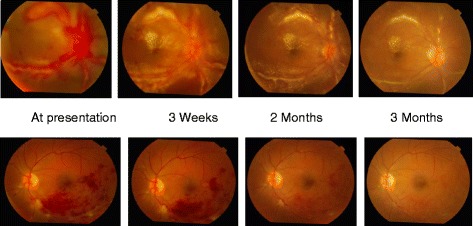
3-month follow up of the patient showing resolution of the lesions. (top) Right eye and (bottom) left eye

At 3 months follow up, the BCVA was 6/9,N8 in the right eye and 6/6,N6 in the left eye. Fundus examination of the right eye showed a hyperemic disc with hard exudates at the macula with resolving exudation and retinal hemorrhages along the vessels in the right eye and the left eye showed resolving retinal hemorrhages along the inferotemporal arcade. In the right eye, FFA showed minimal leakage at the disc with very mild hyperfluorescence along the vessels in the late phase suggestive of resolving vasculitis and the left eye showed no leakage at the optic disc with areas of hyperfluorescence along the vessel walls suggestive of resolving vasculitis (Fig. 2). There was no evidence of neovascularization at the disc or elsewhere. The patient was followed monthly, and there was evidence of gradual decrease in the vasculitis in both the eyes (Fig. 3).

Follow up at 6 months the BCVA in the right eye was 6/9,N8 and in the left eye 6/6,N6. On fundus examination the right eye showed mild pallor of the optic disc with persistence of the hard exudates at the macula and areas of vasculitis with complete resolution of the retinal hemorrhages. The left eye shows resolving retinal hemorrhages along the inferotemporal quadrant with resolving vasculitis.

Follow up at 1 year the BCVA in the right eye was 6/9,N6 and in the left eye was 6/6,N6. Fundus examination of the right eye showed mild pallor of the optic disc with a few exudates at the macula, and the left eye showed formation of collaterals.

## Discussion

Intraocular tuberculosis may present with varied clinical signs and symptoms. Granulomatous uveitis is one of the most common clinical manifestations of ocular tuberculosis [4]. Retinal vasculitis causing secondary retinal vein occlusion is one of the rare manifestations of the disease. However, it is not well understood whether the vasculitis is caused by the infectious agent itself that is mycobacterium tuberculosis or it is an immune-mediated response to the tubercular antigen [5].

On review of literature tubercular vasculitis is said to usually present as peripheral vasculitis and the posterior pole vessels are more commonly involved in vasculitis secondary to a viral etiology. However, our patient presented with a very severe retinal vasculitis at the posterior pole involving all the major branches of the central retinal vein in the right eye and a retinal vein occlusion in the left eye secondary to tubercular vasculitis which is a rare manifestation. Inflammatory etiology is said to be the most common cause for a young patient presenting with retinal vein occlusion [6, 7]. We need to thoroughly investigate the patient for both infectious and non-infectious etiologies. We had two differential diagnosis in mind for our patient—viral retinitis as the clinical picture resembled frosted branch angiitis and IgG was positive for both HSV and CMV [8, 9], and the second was tubercular vasculitis. Frosted branch angiitis has been seen previously is association with systemic tuberculosis [10, 11]. No single test is said to be confirmatory for tuberculosis especially in countries endemic for tuberculosis where the TST is often positive in most of the population secondary to BCG vaccination and frequent exposure [12].

A high clinical suspicion along with strongly positive TST, raised ESR levels, presence of pulmonary nodules, and mediastinal lymph nodes raised our suspicion of tubercular etiology. Confirmation of the diagnosis can be made by histopathological examination of the EUS-FNAC of the mediastinal lymph nodes, which is said to be a safer procedure than transbronchial biopsy [13]. In our case, the histopathological examination of the mediastinal lymph nodes showed necrotic granulomas suggestive of tuberculosis and the patient was treated with anti-tubercular treatment along with oral corticosteroids. One needs to confirm the diagnosis before starting the anti-tubercular treatment, which is a long-duration multidrug treatment with a potential risk of side effects [14].

We report this case to highlight that tubercular vasculitis may have a clinical presentation resembling frosted branch angiitis of viral etiology however one needs to have a strong clinical suspicion and investigate the patient thoroughly including microbiological and histopathological examinations of the samples collected to confirm the diagnosis and provide timely management to salvage the eye.
